# Changes in body composition with a hypocaloric diet combined with sedentary, moderate and high-intense physical activity: a randomized controlled trial

**DOI:** 10.1186/s12905-019-0864-5

**Published:** 2019-12-27

**Authors:** A. Hernández-Reyes, F. Cámara-Martos, R. Molina-Luque, M. Romero-Saldaña, G. Molina-Recio, R. Moreno-Rojas

**Affiliations:** 1 Department of Bromatology and Food Technology, University of Córdoba, Campus Rabanales, ed. Darwin - annex. Office of Dr. Rafael Moreno, 14071 Córdoba, ES Spain; 2 Nursing department, University of Medicine and Nursing of Córdoba, Córdoba, Spain; 3 Department of Occupational Health and Safety, City of Córdoba, Córdoba, Spain

**Keywords:** Motivation, Weight-related goals, Physical activity, Hypocaloric diet, Weight-loss, Aerobic exercise, Dietary adherence

## Abstract

**Background:**

There is evidence showing the effectiveness of a hypocaloric diet and the increase in physical activity on weight loss. However, the combined role of these factors, not only on weight loss but also body composition, remains unclear. The purpose of this study was to investigate the effect of a hypocaloric diet on the body composition of obese adult women throughout different degrees of physical activity during a weight loss program.

**Methods:**

One hundred and seventeen healthy female volunteers were randomly assigned to one of the experimental groups: a control group with a low-level prescription of physical activity (1–4 METs), moderate physical activity group that performed 10.000 steps walking (5–8 METs) and intense physical activity group that trained exercises by at least 70% of VO2max three times a week (> 8 METs). All subjects followed a hypocaloric diet designed with a reduction of 500 kcal/day. Nutritional counseling was provided throughout the study period to help ensure dietary adherence.

**Results:**

We found no differences in body weight compared to moderate and intense physical activity (ßstand. = − 0.138 vs. ßstand. = − 0.139). Body fat was lower in women following an intense activity (ßstand. = − 0.436) than those with moderate exercise (ßstand. = − 0.231). The high-intense activity also increased muscle mass at the end of the intervention, standing out above the moderate activity (ßstand. = 0.182 vs. ßstand. = 0.008).

**Conclusions:**

These findings indicate that a hypocaloric diet, without prescription of physical activity, is adequate to lose weight in the short term (12 weeks), but physical activity is vital to modify the body composition in women with obesity. Body fat was lower when women practiced a moderate exercise compared to hypocaloric diet only, but an intense physical activity was the most effective protocol to obtain a reduction of body fat and maintain muscle mass.

**Trial registration:**

The study protocol complied with the Declaration of Helsinki for medical studies, it was approved by the bioethical committee of Córdoba University, in the Department of Health at the Regional Government of Andalusia (Act n°284, ref.4156) and retrospectively registered in clinicaltrials.gov (NCT03833791). Registered 2 January 2019.

## Background

Adiposity has a negative effect on health, presenting a high co-morbidity with chronic diseases like Type 2 Diabetes and cancer, in addition to causing an increase in general mortality [1]. There are different methods of assessing body fat or weight-related health problems, such as waist measurement. However, the most common method used by experts is the Body Mass Index (BMI), which uses height and weight data to work out if a patient is healthy, overweight, or obese. The negative aspect of BMI is that this index does not provide precise information on body composition, an essential element of evaluating the risk of disease. This aspect has led some authors to define “the obesity paradox” [2] as the situation whereby obese individuals do not appear to be at higher risk than lean individuals of having hypertension, dyslipidemia, type II diabetes, or cardiovascular disease. Recent studies based on the total body fat content argue that adiposity is a significant risk marker to evaluate unhealthy body weight and have proposed this as a more accurate indicator in comparison with the BMI to predict obesity [3]. It is important to note that women are twice as likely to suffer from severe obesity (BMI ≥35 Kg/m2) [4]. Furthermore, the risk increases in postmenopausal women due to the loss of estrogens, which cause an increment in adipose tissue and a decrease of lean body mass [5].

There is considerable evidence regarding the importance of physical activity (PA) in weight loss programs to maintain a healthy weight and, in the long-term, to prevent weight gain [6]. Also, some studies show that an increase in (PA) provides comprehensive health benefits and reduces the mortality rate associated with any cause, regardless of the BMI [7]. Accordingly, specific physical exercise programs should be prescribed to help overweight and obese patients to improve their health. Critical reviews have written whereby the BMI was used as a marker to establish a link between PA and weight loss [8–11].

However, the EPIC-PANACEA study, carried out in Europe with a population of 405,819 subjects, [12] demonstrated the existence of a reverse association between PA, BMI, and abdominal adiposity. There is a consensus whereby, to measure the success of any intervention in programs for modifying the body composition, single indicators like weight loss or BMI should not be used. Evidence has shown that body weight per se cannot be considered to be reliable [13]. Instead, parameters representing the quality of the weight loss, such as muscle mass gain or body fat loss, should be used [14].

We accept that the body fat mass and the fat-free body mass (FFM) will decrease proportionally during a weight loss program [15]. However, when following a diet in combination with PA, the results show that the FFM will not suffer any changes or, in some cases, it will even increase [16]. Therefore, in general, when following a diet with the sole purpose of losing weight, patients may feel disappointed with the results, generating negative feelings such as frustration and deception. As indicated elsewhere, [17] a better understanding is needed to design more practical interventions based on evidence. To achieve this, educational activities, together with a diet and a PA program, will help the patient understand the benefits of weight loss on overall health.

An intervention proposal that includes dietary adaptations and a PA program with a net caloric balance close to zero will result in no changes (or minimum changes) in the body weight of individuals with excess weight. Nevertheless, the intervention will provoke a reduction in BF compensated by an increase in FFM [18]. Among the mechanisms that would explain the scant difference in weight loss between people following a diet or a diet/PA combination, other authors suggest that; 1) patients increasing adaptation to the physical activity program, which implies a lower energy expenditure over the time [19]; and 2) that there is an increase in appetite as the energy expenditure increases due to PA [20].

In summary, this research aims to evaluate the impact of different physical activity levels on the BF, FFM and body weight in adult women with excess weight or obesity that have followed the same dietary pattern.

## Methods

### Subjects

The sample was composed of 117 Caucasian healthy adult female volunteers from the area of Andalucía (Spain) (age: 42.97 ± 10.84 yrs.; height: 161 ± 0.07 cms; Weight: 82.56 ± 14.46 kgs). All patients were recruited from two private clinics, to which women went to lose weight.

The sample size was calculated using Fleiss equation, for a power of 80%, a two-sided significance level of 95% and expecting that 5% of the women who do not receive exercise intervention lose weight while this figure will reach 40% in those receiving exercise prescription (moderate or high dose). Although sample resulted in 51 individuals (17 women per group: non-exercise, moderate and a high dose of exercise), a size of 60 women (20 for each group), was finally estimated, to mitigate the effect of possible losses during this trial.

All the participants reported that they did not perform any particular physical activity, so they categorized as sedentary. The initial study required adulthood who classified as overweight or obese according to the body mass index (BMI, calculated as kg / m2) of ≥25) and that they should not be taking a diet to lose weight at the time of the interview or have been on a diet in the 6 months prior to recruitment. People who met these criteria were invited to attend a familiarization session, lasting approximately 1 h, on an individual basis with a dietitian-nutritionist. In this session, patients provided information about how to carry out the diet, as well as pedagogical information about the healthy lifestyle and food. At this time, a complete medical history and informed consent recorded.

**Fig. 1:**
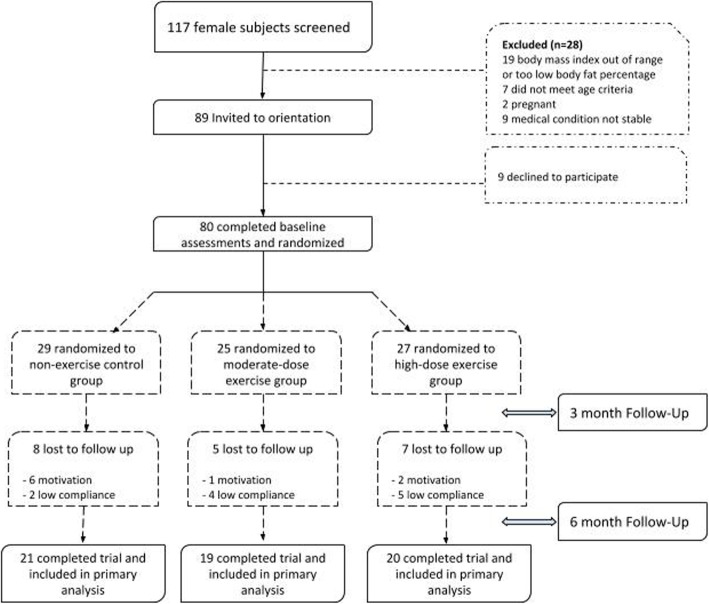
CONSORT flow diagram

Women with the following pathologies or special situations were excluded from the study: Type II diabetes, being or trying to be pregnant, being in a maternal lactation period, suffering from kidney failure, being underage, presenting healthy weight (BMI ≤ 25) or receiving anti-depression pharmacological treatment. On the other hand, the inclusion criteria were: having a body fat percentage ≥ 30, being sedentary, and not having been submitted to a restrictive diet in the 6 months before the beginning of the study. The flow chart of the participants is shown in Fig. 1. Those women who met the eligibility criteria were scheduled for initial body measurements.

This study was approved by the bioethical committee of Córdoba University, in the Department of Health at the Regional Government of Andalusia (Act n°284, ref.4156).

### Subject randomization

After obtaining the written informed consent, and completing initial screening tests, subjects were assigned to one of three groups: control, moderate physical activity, and intense physical activity group, through standard computer-based procedures (random number generator). After completing the tests, subjects were randomly assigned to 1 of 3 experimental groups: a control group that only follows a hypocaloric diet without prescription of physical activity (CON) (*n* = 29) or a moderate training group (MPA) that in addition to supporting the same diet, is assigned to perform moderate physical activity (*n* = 25) and a group of intense physical activity (IPA) (*n* = 27).

### Testing sessions

The initial assessment was carried out in the 24–48 h before the beginning of the intervention. From this moment, an appointment was given to each patient. The follow-up during the time of the study was carried out after the overnight fast and always on the same day and time of the week in order to minimized variability between sessions. The initial evaluation included a completed anthropometric study: height, total body weight, body fat (BF), muscle mass (MM), and body water. These parameters were all recorded in every weekly session together with the PA data.

### Dietary intervention

Subjects were provided with customized dietary plans designed by an experienced nutritionist (A.H.R). The daily energy requirements were determined by estimating the energy expenditure as previously reported Harris-Benedict [21]:

$$ \Big(655.0955+9.5634\ \left[\mathrm{Weight}\ \left(\mathrm{kg}\right)\right]+1.8496\ \left[\mathrm{Height}\ \left(\mathrm{cm}\right)\right]-4.6756\ \mathrm{Age}\ \left(\mathrm{in}\ \mathrm{years}\right). $$

A multiplier factor of 1.5 was applied to the value resulted in the equation in those patients carrying out physical activity [22]. During a period of 24 weeks, all the patients followed a diet with the following distribution of macronutrients: 25–30% proteins, 40–45% carbohydrates, and 30–35% fat. The moderated-fat restricted-calorie Mediterranean diet is rich in vegetables and low in red meat, with poultry and fish replacing beef and lamb, with a goal of no more than 35% of calories from fat. This diet is based on the recommendations of Willett and Skerrett [23]. The diet was hypocaloric with a reduction of 500 kcal/day during the treatment period to achieve a weekly weight loss of 400 g, an amount that is a safe, achievable, and clinically meaningful goal for weekly weight loss [24]. Dietary protein intake was set at 1.8 g/kg of body mass, as higher protein consumption has been shown to help offset losses in lean tissue mass and promote greater adherence to the nutritional regimen (REF) [25]. No vitamins or other nutritional complements were prescribed. After being included in the study, each woman participated in a 1-h seminar in which the Dietitian-Nutritionist instructed them on how to make a suitable selection of food and prepare it. The menu proposed was valid for 1 week, and a new diet was provided to each woman in the weekly follow up appointment. The energy and nutritional intake were evaluated by the program Dietowin® and the weighing method (Dietowin 8.0, 2015) [26]. Continued nutritional guidance was provided to the subjects at the time of each training session by the research team to encourage dietary adherence.

### Exercise training intervention

To estimate the degree of physical and sedentary activity at the beginning of the study, we used the extended version of the International Physical Activity Questionnaire (IPAQ-long), which is reliable and valid for estimating physical activity and sitting-down time [27]. To adjust for sedentary behavior and physical activities outside working hours, the IPAQ-long was interview-administered at the beginning of the study and repeated at the end of the intervention. For the PA, the strata proposed by Matthews were applied [28]. A pedometer was installed on the mobile phones of all patients (ACCUPEDO). Accupedo uses the phone’s built-in accelerometer in its algorithm. The application is designed to work regardless of whether the phone is placed. Each woman was carefully informed about how to wear the device during walking hours. The app recorded all the steps executed during the day without user intervention. The caloric cost of physical activity was calculated based on the one metabolic equivalent (MET) criteria, defined as the amount of oxygen consumed while sitting at rest with a value of 3.5 ml O2 per kg body weight x min. The MET concept represents a simple, practical, and qu ickly understood the procedure for expressing the energy cost of physical activities as a multiple of the resting metabolic rate [29].

Patients were allocated in one of three different training groups.

The CG was provided with information related to daily recommended PA levels and information about the benefits of walking regularly at least 30 min daily; approximately 5000 steps can be reached during this training session. This level of physical activity can be considered sedentary or o low level, that requires minimal movement or energy expenditure (1–4 metabolic equivalent units; METs) and is associated with sitting or lying down during walking hours [30]. Patients in the MPA were provided with the same information as the CG about the benefits of walking. This group was prescribed to achieve a goal-setting of 10,000 steps per day and were informed that this value was equivalent to 60 min of walking per day, that they had to reach a moderate-vigorous rate, around 60% of VO2max [31]. Their heart rate (HR) was calculated using the Karvonen formula, [32] and the maximum HR was determined by the formula: 220 – age (years). This level of physical activity has a rate of 5–8 units of METs. For the IPA, the patients had to training sessions of intense physical activity with an intensity between 60 and 80% of maximal muscle strength three times a week [33]. During the 24 weeks lasted the study, the participants in the IPA group were prescribed three BodyPump sessions weekly supported by free access to fitness club offering this exercise during the intervention period.

BodyPump is a pre-choreographed and strengthening workout session. Each session includes strength exercises targeting specific muscle groups, and the participants exercise with a weight bar (1.25 kg), plates (1, 2.5 or 5 kg), and a step. Each session includes between 800 and 1000 repetitions in the range of 50–100 repetitions in each muscle group. There are 1–2 min rest periods between each track, used to change weights and prepare for the next exercises [34]. Women did not walk the BodyPump days, but the rest days of the week, they had to walk 1 h at the same intensity as the MPA group, to unify the volume dedicated to the exercise in each group. The IPA group had a substantial caloric cost of more than 8 METs.

The follow-up tests began during the first week of the diet and physical activity assignment. The body composition was measured following an overnight fast, and the subject was required to go to the center on the same day of the week, at the same time, and to wear the same clothes. Review appointments continued weekly until week 24 when all the variables were collected.

### Anthropometrics and body composition measurements

Body fat percentage (%BF), MM and the percentage of water (%W), were considered as being result variables, and were monitored and recorded throughout the time by multifrequency electrical impedance (BWB-800A, Tanita Corp. USA), previously validated [35]. This method is based on a 3-compartment model capable of evaluating BF, MM and bone mineral content. Also, the percentage difference of each dependent variable collected in the control consultations were recorded and compared to that in the first consultation, to evaluate the changes produced. The independent variables recorded were: age (years), height (cm), weight (Kg) and BMI (Kg/m^2^).

The anthropometric measurements were taken following the recommendations of the standardized anthropometry handbook, [36] and by professionals in order to reduce the coefficient of variation. Each measurement was taken at 3 different times, calculating the mean value. All the quantitative variables were measured with a precision of 0.1. For the height, a stadiometer was used (SECA 213).

### Statistical analyses

The quantitative variables have been presented with mean and standard deviation, whereas the qualitative ones in frequencies and percentages. To contrast, the goodness of fit to a normal distribution of data coming from quantitative variables, the Kolmogorov-Smirnov test corrected by Lilliefors, was employed. To compare the bivariate hypothesis, the 2-means Student’s t-test was applied, while for the qualitative variables, the Chi-square test was used, and the Fisher exact one when necessary. Likewise, for the analysis of three or more means, the ANOVA repeated means test was employed to evaluate the effects of the intervention at the baseline moment, and 3 and 6 months, and the correlation between the quantitative variables was checked by the Pearson (r) coefficient of correlation. Finally, in the case of not meeting the normality or homoscedasticity criterion, the non-parametric versions of the above tests were made.

Adjusted linear regressions were made for each variable of the body composition (%BF and MM) and the weight at the end of the study to estimate the Beta standardized coefficients of the physical activity in achieving the goals. To determine the goodness of fit of the models, the standard error, the adjusted coefficient of determination, the F statistics, the analysis of the linearity, and the residues were analyzed.

For all the statistical analyses, an alpha error probability of under 5% (*p* < 0.05) was accepted, and the interval of confidence was calculated as being 95%. For the statistical analysis, the computer programs IBM SPSS Statistics version 22.0 were used.

## Results

The women included in the study were of a mean age 42.97 ± 10.84 (BI95%: 40.17–45.77). With respect to their body composition, in the first consultation, a mean weight of 82.56 ± 14.46 Kg (BI95%: 78.83–86.30 Kg) was found, a body fat percentage of 42.17 ± 5.50% (BI95%: 40.75–43.59%); and a muscle mass of 44.71 ± 5.08 Kg (BI95%: 43.40–46.02 Kg). There were no significant differences in the baseline data between the physical activity groups to which each woman was randomly assigned (Table 1).

**Table 1:**
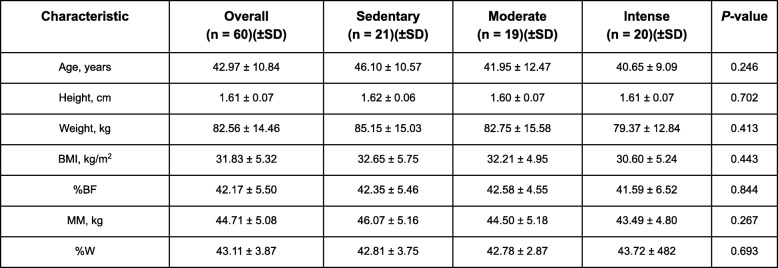
Participant characteristics at baseline

### Analysis of related means in the modification of the body composition at 3 and 6 months of intervention

At 3 months of treatment a significant reduction in the body weight with respect to the baseline measurements was observed, with the mean of 82.56 ± 14.46 Kg (BI95%: 78.83–86.30 Kg) dropping to 76.91 ± 12.94 Kg (BI95%: 73.57–80.25 Kg) (*p* < 0.001). This trend was maintained in the rest of the anthropometric variables, producing a decrease in the BMI, in the %BF and in the MM. On the other hand, the %W rose from a mean of 43.11 ± 3.87% (BI95%: 42.11–44.11%) to 44.90 ± 4.14% (BI95%: 43.81–45.97%) (Table 2).

**Table 2:**
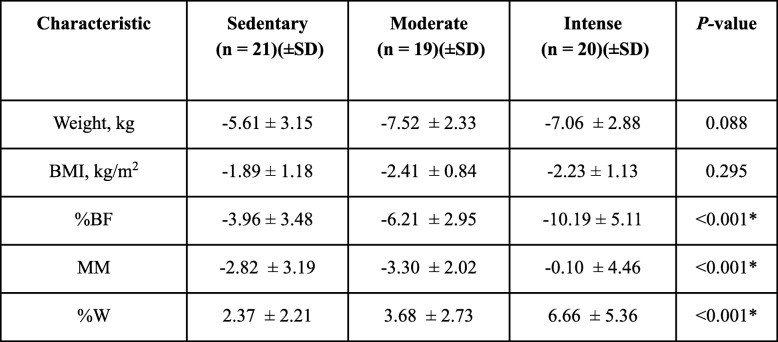
Changes in body measurements at 3 months

Similarly, at 6 months, and globally, the participants showed statistically significant variations both with the measurements obtained in the first nutrition consultation and with those taken at 3 months. In this sense, a diminution in weight of the BMI (*p* < 0.001), in the %BF, and an increase in the %W (*p* < 0.001) were observed. However, although the MM exhibited a significant drop between the first moment and at 3 months (*p* < 0.001), there were no differences (*p* > 0.05) between those found in the controls at 3 and 6 months.

### Analysis of the evolution of the body composition in terms of physical activity

The analysis of the variation in the body composition in the different PA groups was made based on the percentage modifications between the measurements collected in a basal manner at 3 and at 6 months.

#### Changes at 3 months of monitoring

At 3 months of monitoring, no significant differences were found between these three activity groups with regard to that referring to a reduction in weight or in the BMI. With respect to fat, a greater decrease in the IPA group compared to the other two groups (*p* < 0.01) was observed. However, the fat loss among those who followed a sedentary lifestyle and moderate physical activity did not display significant differences (*p* = 0.204). As for the MM, the value of the latter fell notably in the above group of women, and the difference found (*p* = 0.204) was not significant. However, the reduction in the IPA group was less marked, the difference being significant with respect to both groups (*p* < 0.05) (Table 3).

**Table 3:**
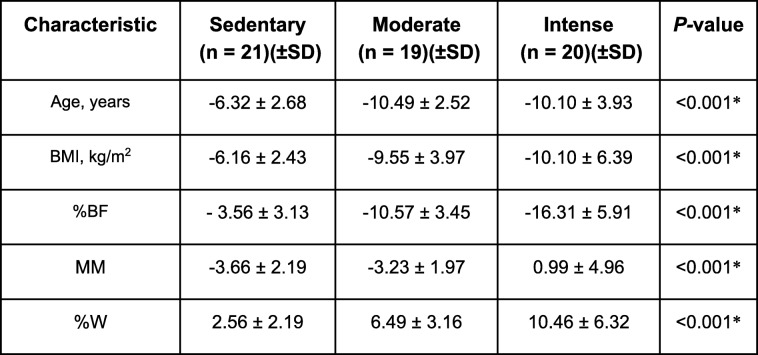
Changes in body measurements at 6 months

#### Changes at 6 months of monitoring

The trend shown at 3 months was maintained at 6 months in all the anthropometric variables. As for weight, a marked fall in weight was found in those patients who carried out some type of moderate or intense PA compared to the sedentary ones (*p* < 0.001 and *p* < 0.003, respectively). However, the weight did not significantly vary between the groups doing some type of physical activity (*p* = 0.976). Similar behavior was observed in the BMI modification.

9pt?>The %BF decreased to a greater degree (− 16.31 ± 5.91 (BI95%: − 19.08 - -13.54), *p* < 0.001) in the most active women, this being the only activity group in which MM was gained (0.99 ± 4.96 (IC95% -1.33 – 3.31), *p* < 0.001), with no significant differences between those who did not do any physical activity or did a moderate kind (*p* = 0.793) (Table 4).

**Table 4:**
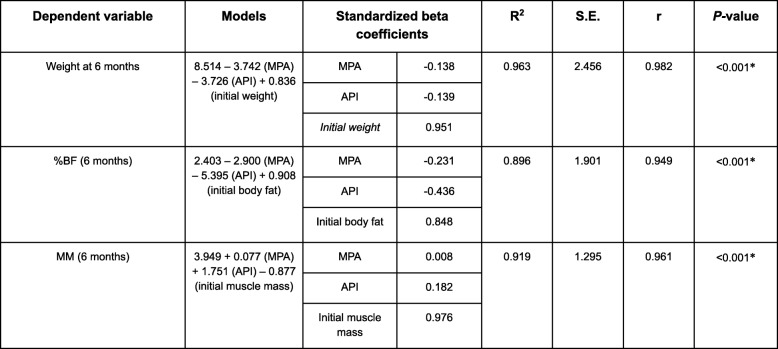
Multiple regression models

### Role of physical activity intensity in body composition modification

The body composition study at 6 months revealed that the impact of PA varied depending on which parameter of the anthropometric evaluation was analyzed. Thus, it was decided to conduct a multivariant analysis by means of a multiple linear regression in order to obtain fit models that would determine the effect of the physical activity (sedentary, moderate, intense) on each of the anthropometric variables through the calculation of standardized Beta coefficients.

In the fit models (Table 4) it is seen how 96.3% of the weight variability at 6 months is explained by the initial weight and the physical activity done, with no differences found between carrying out one that is moderate or one that is intense (ß_stand._ = − 0.138 and ß_stand._ = − 0.139, respectively).

However, the moderate and intense physical activities behave differently in the BF modification and in the MM.

**Fig. 2:**
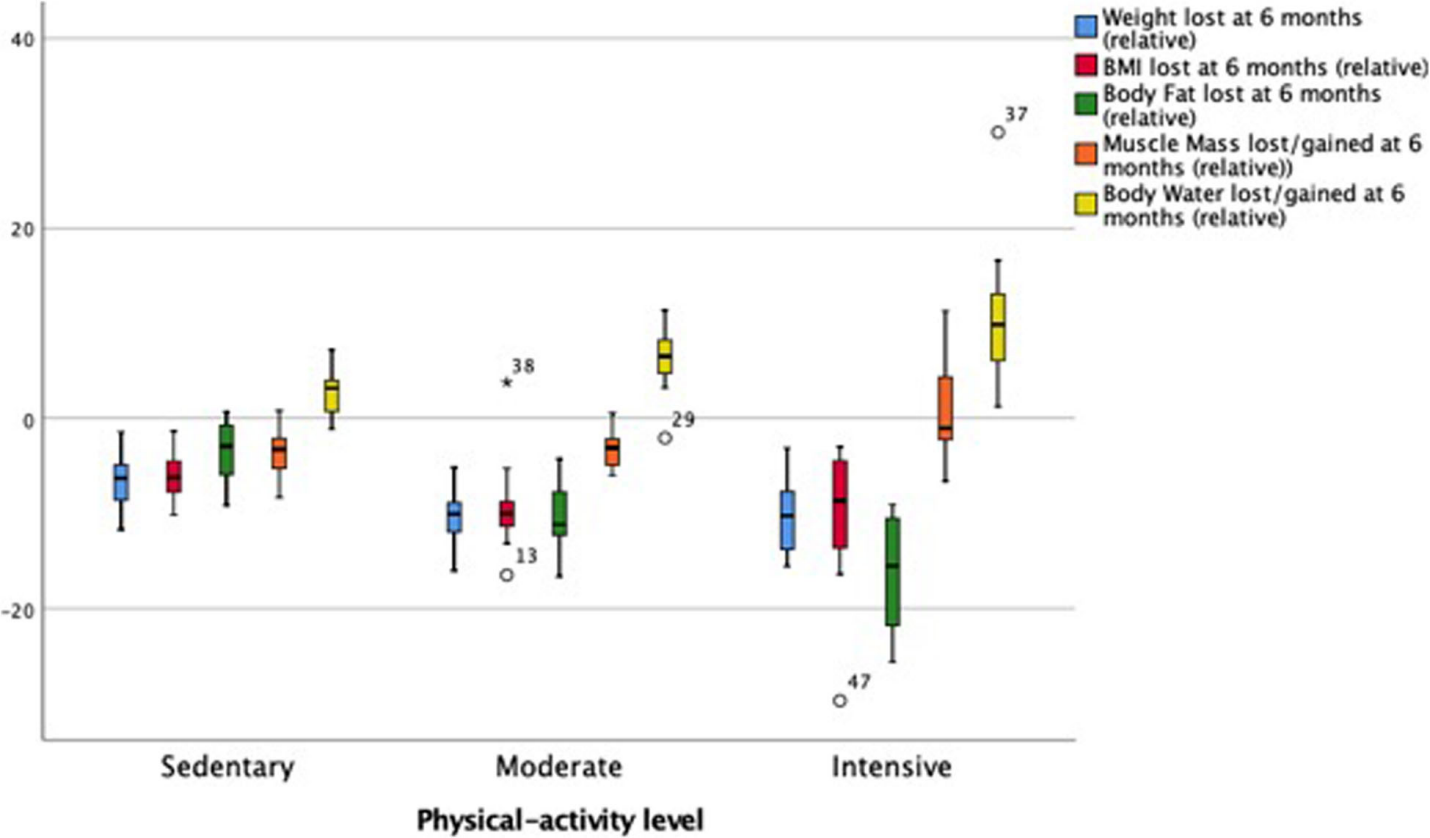
Modification of body composition at 24 weeks depending on the physical activity prescribed in combination with hypocaloric diet

With regard to BF modification, the intense activity had a greater capacity (practically twofold) of diminishing BF at 6 months (ß_stand._ = − 0.436 vs. ß_stand._ = − 0.231). This degree of PA also exerted a greater effect on the increase in MM at the end of the intervention, standing out above the moderate activity (ß_stand._ = 0.182 vs. ß_stand._ = 0.008) (Fig. 2).

## Discussion

### Summary (overview)

This study aimed to assess the effect of varying intensity of physical activity on body composition during an intervention period of 6 months. The findings show that, when incorporating an MPA program based on aerobic exercises to a dietary regime, weight loss improves significantly. Also, women’s body composition shows a definite improvement. In this case, MPA raised the BF loss to − 6.21% (± 2.95%) after 3 months, and to − 10.57% (± 3.45%) after 6 months. These losses are significantly higher than those in the control group. However, the most significant fat loss was achieved by the group performing IPA, which showed a decrease of − 16.31% (± 5.91%) after 6 months. It also found that, after a caloric restriction (CON), the loss of MM reached up to − 3.66 ± 2.19 kg after 6 months.

The intervention showed that the best intensity for carrying out PA is the intense one, due to its influence on body composition. After 3 months, the MM loss was significantly less than for the other groups (− 0.10 ± 4.46 Kg). More importantly, after 6 months, the group doing IPA was the only one that gained MM (0.99 ± 4.96 Kg). Therefore, it is of interest to observe how a combination of aerobic and anaerobic training permits, not only to conserve but also to increase MM, which suggests that the weight loss triggered by the IPA is of better quality than that produced by the rest of the interventions studied. In short, the main results obtained demonstrate that weight loss is significant in the short-term (3 months), when consuming a hypocaloric diet (with an energy deficit of 500 kcal/day), with no need of PA. However, weight loss in the mid-term (6 months) will depend on incorporating measured and controlled physical activity, irrespective of its typology or intensity (moderate or intense).

Finally, it manifest that the body composition changes in the short term when a diet and a physical activity plan are followed (independently of the exercise intensity). It is only through intense physical activity that a reduction in the BF can achieve, together with maintenance or increase in FFM. These two parameters are of great importance to evaluate the quality of the weight loss.

### Comparison with existing literature

Concerning body weight, it has been shown that a one-off nutritional intervention may trigger a moderate loss of weight (from 5 to 10%) in middle-aged adult women, and in post-menopausal women ≥50 years, in comparison with their initial weight [37]. These data are in agreement with the results presented in this study, in which a weight reduction of 6.32 ± 2.68% found in the control group after 6 months.

Other works focusing their analysis on the effectiveness of PA together with caloric restrictions [38–41] conclude that the difference in weight loss in the group only following a diet is moderate (from 1 to 2 extra kg). These results are similar to those found after the present intervention, in which there were no significant differences in weight loss in the study groups after 3 months.

Contrary to findings presented by other authors [42], no sustainable weight loss can ensure in adult women only with negative caloric balances mediated by a caloric restriction after 3 months. Furthermore, after 6 months, the weight loss amongst the women who only followed the nutritional intervention was significantly less, in comparison with the women with a PA and a diet plan. These differences at 6 months, compared with those founds in previous works, could be due to variations in the study design. All groups, regardless of the PA prescribed, were equally submitted to a negative caloric balance induced by a caloric restriction of 500 kcal/day. This enabled evaluation of the total body weight evolution with greater precision, unlike other studies in which the energy balance was not rigorously controlled [43, 44].. Also, there is evidence that women, in contrast to men, show a greater need to compensate their appetite after a caloric restriction, and that this compensation is not closely related to doing acute exercise [45]. Therefore, we consider that monitoring and controlling the dietary intake allows us a better understanding of weight loss evolution in women.

During the intervention, it found that the MPA group achieved the most considerable weight reduction during the first 3 months, a trend that continued in the second term. This finding confirms the conclusions of Mayer in the 1950s, in which the activity intensity described as “range of normal activity,” and which was recently redefined by Blundell et al. as a “regulation area.” [46] Also, we know that a lower level of energy expenditure (CON) may imply a disconnection between intake and caloric expenditure [47]. PA performs an essential part in regulating satiety. This research has shown that a moderate-intensity exercise program helps patients reach higher levels of satiety than those with a sedentary lifestyle, thus representing the ideal PA threshold for losing weight.

The MM gain has explained the smaller weight loss produced by doing PA. Similar to findings from other research, and this is due to strength exercise being a stimulant of growth and muscular maintenance in adult women, absorbing the weight loss [48–50].

Focusing the analysis on body composition, the women submitted to caloric restriction lost their BF for a limited time. After 3 months, BF loss in the control group was a mean of − 3.96% (± 3.48%), corroborating with results from other studies [51, 52]. However, at 6 months, there was a recovery of BF in this group, with an overall reduction at the end of the intervention of − 3.56% (+ 3.13%).

The explanation for this could be found in the aforementioned “regulation area,” that relates adipose tissue accumulation to the small amounts of energy expended. Regular exercise at a tolerable threshold acts as an appetite modulator and, as other investigations have shown, in a reduced PA scenario the patient tends to eat in excess or an opportunistic manner [53, 54].

The loss of muscle mass after caloric restriction (CON) that reached up to − 3.66 ± 2.19 Kg at 6 months was less pronounced amongst women who carried out MPA, mitigating it to − 3.23 ± 1.97 Kg at 6 months, with a slight recovery of the MM lost at 3 months (− 3.66 ± 1.97), similar to that reported by other authors [55].

These results are reaffirmed in the multivariant study. Following the same caloric restriction dietary pattern, the changes in body composition will depend on the type of physical activity. Here, intense physical activity achieved better results in BF loss (ßstand. = − 0,436) and a gain in, or maintenance of, MM (ßstand. = 0,182) and confirms the findings of other authors (Friedenreich et al., 2011). Provided it is possible, intense physical exercise should be recommended in this group of women.

From this perspective, it is essential to emphasize the importance of losing weight correctly, as defined by a reduction in body fat percentage with no impact on the MM. Meanwhile, a loss of MM causes an increase in the risk of suffering from sarcopenia [37]. That is to say, a quality weight loss would enjoy the benefits of BF loss without the risks of MM loss. Besides, evidence has shown that PA improves behavior patterns that *prevent* the rebound effect once the controlled intervention has ended [56].

### Body composition

Previous studies reported that decreases in the MM were restored overtime after weight loss interventions [57, 58]. Although the MM loss was lower than that of total BF, muscle maintenance must be monitored and prescribed even in weight loss programs. The subjects of our study who had a sedentary or moderate exercise prescription, that is, walking, lost muscle mass at the end of the period. The explanation for this is that this kind of PA seems insufficient for mobilizing and stimulating muscle mass [59]. The group in our study that had an intense physical exercise prescription, i.e., incorporating resistance training, was the only one that showed a mass muscle gain at 6 months. These results might evidence that a combined program of aerobic and resistance-type exercise helps to preserve muscle mass during weight loss, results that matched those of a recent review [60].

Our findings reveal a significant body fat loss in the prescription of PA; the higher the intensity of PA, the greater the loss of fat at 6 months. While sedentary instruction implied a 3% fat loss at 6 months, the IPA subjects reached 16%. Also, when analyzing the results among MPA and IPA subjects, we observed that they lost an additional 6% of fat, while we did not find a significant weight loss. Our results are consistent with the existing literature [61]; although high-intensity training did not improve the weight loss in an interval of 6 months compared to a lower intensity, the impact on the fat loss was significant [62].

### Strengths and limitations

For this clinical study, the sample size was 60 participants. All of the patients were in a similar baseline in order to undertake a better assessment at the end of the research. To evaluate the changes and the importance of the physical activity on the body composition, the study was based on an exercise plan with two intensities (moderate and intense). The study has limitations because the sample is limited to sedentary, overweight or obese, adult women. Thus, further investigations should be necessary to clarify if these results could extrapolate to men. Although the sample size is similar to that used in previous works [63, 64], we carried out a randomization procedure that led to balanced arms. Also, a 20% attrition rate would expect at 6 months, and the study saw a slightly higher rate of 25%. While attrition was more significant than was intended, it is similar to observed in several other physical activity weight-loss interventions [65, 66]. No differential attrition rates were found between groups in the present study. To avoid the self-report bias, previously documented [67], the data collection records check out by the research staff in face-to-face consultations weekly. A control without caloric restriction was not added to this study because the effect of the diet was not the primary objective to design this investigation, using as a control group the sedentary one. To further investigate how diet could affect behavior during a weight loss program, a non-restricted group might be interesting.

Although a period of 6 months is considered to be adequate to draw meaningful results and valuable conclusions, future studies with longer monitoring periods will be beneficial to confirm whether the trend regarding the MPA and IPA has been maintained.

Lastly, it will also be beneficial for further research to include a fourth group with no diet assigned. This will help to assess whether body composition can improve in the short, medium and long term with only PA.

## Conclusion

Although it is common to prescribe a hypocaloric diet to lose weight, this strategy is only useful for a short period. It postulated in this study that a combination of PA and controlled caloric restriction achieve a suitable modification of body composition in overweight or obese women. From the different levels of PA investigated herein, it found that an intense level (IPA) was the most effective for promoting body fat loss with a simultaneous maintenance/gain in MM. Therefore, provided that the patient is fit to participate in IPA, this should be recommended for a weight loss program. Furthermore, moderate-intensity PA, although less effective at mitigating MM loss than IPA, was found to guarantee a more significant decline in body fat than that obtained by a one-off caloric restriction. These findings are of interest to the Public Health sector, given that they have highlighted that occasional caloric restriction strategies in overweight or obese women will not be sufficient for achieving mid- or long-term health objectives.

## Data Availability

The datasets used and/or analyzed during the current study are available from the corresponding author on reasonable request.

## Abbreviations

%BF: Body fat percentage
%W: Percentage of water
BF: Body fat
BMI: Body mass index
CON: Control group
FFM: Fat-free mass
HR: Heart rate
IPA: Intense physical activity group
MM: Muscle mass
MPA: Moderate physical activity group
PA: Physical activity
